# High ion conductivity based on a polyurethane composite solid electrolyte for all-solid-state lithium batteries

**DOI:** 10.1039/d1ra07971a

**Published:** 2022-01-31

**Authors:** Peng Cui, Qi Zhang, Chun Sun, Jing Gu, Mengxin Shu, Congqiang Gao, Qing Zhang, Wei Wei

**Affiliations:** College of Electronic and Optical Engineering & College of Microelectronics, Nanjing University of Posts & Telecommunications 9 Wenyuan Road Nanjing 210023 Jiangsu China weiwei@njupt.edu.cn

## Abstract

Solid polymer electrolytes (SPE) are considered a key material in all-solid Li-ion batteries (SLIBs). However, the poor ion conductivity at room temperature limits its practical applications. In this work, a new composite polymer solid electrolyte based on polyurethane (PU)/LiTFSI–Al_2_O_3_–LiOH materials is proposed. By adding a few inert fillers (Al_2_O_3_) and active agents (LiOH) into the PU/LiTFSI system, the ion conductivity of the SPE reaches 2 × 10^−3^ S cm^−1^ at room temperature. Exploiting LiFePO_4_ (LFP)‖Li as electrodes, the PU-based composite lithium battery is prepared. The experimental result shows that the LFP|SPE|Li displays high specific discharge capacity. The first specific discharge capacities at 0.2C, 0.5C, 1C and 3C are 159.6, 126, 110 and 90.1 mA h g^−1^ respectively, and the Coulomb efficiency is found to be stable in the region of 92–99% which also shows a desirable cyclic stability after 150 cycles.

## Abstract 2

Adding inert filler to reduce the coupling effect of functional groups to Li^+^ and modifying the functional groups to reduce the adsorption of Li^+^ can lead to high ionic conductivity.

## Graphical abstract

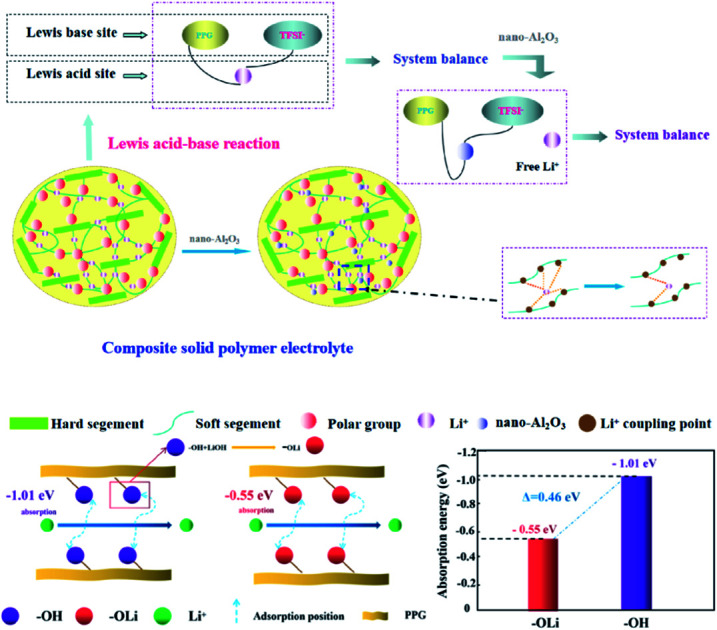

## 1 Introduction

As the core of all-solid-state batteries, solid-state electrolytes have been paid adequate attention for their advantages over traditional liquid state electrolytes.^1–3^ Among all types of solid electrolytes,^4–7^ polymer electrolytes have become the focus due to their excellent mechanical properties and molecular modification.^8^ However, their poor ion conductivity at room temperature seriously restricts the use of solid-state lithium batteries (SSLBs).

At present, numerous methods have been exploited to improve the ion conductivity of the solid polymer electrolyte, such as introducing active fillers and inert fillers.^9^ Lithium salts, such as LiTFSI, γ-LiAlO_2_,^10,11^ and LiN_3_,^12^ are generally used as active fillers because they can directly provide Li^+^ to the polymer system. Inert fillers such as TiO_2_(ref. 13), ZrO_2_,^14^ and Al_2_O_3_(ref. 15,16) can increase the ion conductivity of the system by reducing the polymer crystallinity or coupling of the polymer chain to Li^+^.^16,17^

Polyethylene oxide (PEO)/Li^+^ has been an extensively studied polymer electrolyte system because of its flexibility, inexpensiveness, light weight and high Li^+^ conductivity in SPEs.^18^ However, its inherent softness obstructs the effect of suppressing Li dendrite propagation, which prohibits its applications in Li-ion batteries (LIBs).^19,20^ In contrast, polyurethane (PU) shows not only a good ability to dissolve a large amount of lithium salts but also an excellent stress–strain properties, which compensates the shortcoming of PEO/Li^+^.

PU as a kind of elastic materials is composed of the “soft segment” unit and the “hard segment” unit by the reaction between polyether polyol and isocyanate. PPG (octahydroxy sucrose-oxide allyl ether) which works as a component of the soft segment in PU structure, shows a good ability to dissolve lithium salts.^21–23^ Meanwhile, the hard segment of phenyl, carbonyl, and amide groups in PU can provide good mechanical properties for electrolytes. Chen *et al.*,^21^ designed a waterborne polyurethane, and its conductivity was only 5.44 × 10^−6^ S cm^−1^ at 40 °C. Shibat *et al.*,^24^ prepared a electrolyte which has a conductivity of 10^−5^ S cm^−1^ at room temperature by using polyether polyurethane, and polysiloxane. So far, there have been many reports based on PU/Li^+^-based composite electrolytes, although they^21,25^ have good conductivity at high temperatures (>60 °C), high conductivity (10^−3^ to 10^−4^ S cm^−1^) is also required at room temperature and low conductivity (10^−5^ to 10^−6^ S cm^−1^) at ambient temperature^26–28^ restricts their applications in LIBs.

In this work, we propose a novel composite polymer electrolyte (PU/LiTFSI–Al_2_O_3_–LiOH). Herein, polyether polyols (octahydroxy sucrose-oxide allyl ether) are selected as a “soft segment” backbone “R–O–R” of PU due to the fact that they are rich in hydroxyl groups (–OH), which can control the cross-linking degree and carry out the further modification. Diphenylmethane diisocyanate (MDI) is selected as a hard segment backbone by reacting with PPG in order to form “–NH–C=O” and “–O–C=O” groups. Lithium salts (LiTFSI) and nano-γ-Al_2_O_3_ are used as active fillers and inert fillers respectively. Lithium hydroxide (LiOH) is used as a functional modifier. In order to obtain higher ion conductivity, the effects of the Li^+^ content, nano-acid-Al_2_O_3_ addition, and reaction between LiOH and PPG on the conductivity of the composite electrolytes are investigated. Theoretical calculation is used to study the effect of the change of functional groups on the ionic conductivity, and the EIS AC impedance and assembled battery are used to evaluate the specific charge/discharge capacity and electric cycle stability at room temperature.

## 2 Experimental section

### 2.1 Preparation of the PU-based composite electrolyte

#### 2.1.1 Materials

Polyether polyols (PPG, octahydroxy sucrose-oxide allyl ether, hydroxyl value: 450, *M*_w_: 580–600), LiTFSI (bisfluoromethane sulfimide lithium, C_2_F_6_LiNO_4_S_2_, 99.99%), lithium hydroxide monohydrate (LiOH·H_2_O, 99.995%), acidic-nano-Al_2_O_3_ (99.9%, *d* = 5–10 nm), and diphenylmethane diisocyanate (MDI, 98%, C_15_H_10_N_2_O_2_), all raw materials, are provided by Aladdin, China, and directly applied without undergoing further purification process.

#### 2.1.2 Preparation of PU/LiTFSI (SPE 1)

First, PPG 22 mL (20 g, 0.03 mol) was added to a 50 mL beaker, followed by heating to 90 °C with stirring. Then, LiTFSI 1 g (0.003 mol) was added and stirred for 2 h until completely dissolved. Finally, 0.1 mL PPG/LiTFSI and 0.01 mL MDI were added to a mold of button cell shell of CR2016 to form PU at room temperature.

#### 2.1.3 Preparation of the PU/LiTFSI–Al_2_O_3_ composite electrolyte (SPE 2)

0.05 g (0.0004 mol) Al_2_O_3_ was added to the above (PPG/LiTFSI) system and stirred for 2 h until there was no white substance in the solution. Take 0.1 mL PPG/LiTFSI–Al_2_O_3_ and 0.0125 mL MDI (PPG_mol_ : MDI_mol_ = 2 : 1) and react with each other to form the PU/LiTFSI–Al_2_O_3_ composite electrolyte in a mold of button cell shell of CR2016 at room temperature.

#### 2.1.4 Fabrication of the PU composite electrolyte [PU/LiTFSI–Al_2_O_3_–LiOH]

PPG 20 g (0.03 mol) was added into a 50 mL beaker, followed by heating to 90 °C with stirring. 5 g (0.1 mol) LiOH·H_2_O was dissolved with 5 mL deionized water for the preparation of the stationary aqueous solution at a concentration of 1 g mL^−1^. Then the LiOH solution was added to beaker to react with PPG and heated to 130 °C. After the reaction was finished, the stirring was continued for 36 hours under 130 °C to remove water (noting: in this step that the water must be removed, otherwise the PU cannot be formed with the MDI). Then, LiTFSI 1 g (0.003 mol) and 0.05 g (0.0004 mol) Al_2_O_3_ were added and stirred for 2 h until completely dissolved. Finally, 0.1 mL PPG/LiTFSI–Al_2_O_3_–LiOH and 0.01 mL MDI (PPG_mol_ : MDI_mol_ = 2 : 1) were added to a mold of button cell shell of CR2016 to form PU at room temperature. The sample was then dried in a vacuum oven at 120 °C for 48 h (the operating loss error of this step is 0.2%).

### 2.2 Structure characterization

SPE morphology was observed using the field emission scanning electron microscope (S4800). XRD patterns were documented by using the X-ray diffractometer (Bruker AXS D8 Advance), with Cu Kα radiation (*λ* = 1.5406 Å) over the range of 2*θ* = 3.0–50.0°. The FTIR spectra were obtained by using the Fourier transform infrared spectrophotometer (PerkinElmer Spectrum Two). The TGA/DSC data were documented by using Mettler DSC3, with temperature range: 10–1000 °C, heating rate of 10 K min^−1^, under N_2_. The stress–strain property was evaluated by using the ZQ-990 series universal testing machine. All samples for evaluation have a dimension of 20 mm (*W*) × 50 mm (*L*) × 0.035 mm (*H*).

### 2.3 Battery assembly and measurements

Ionic conductivity measurements based on alternating current impedance spectroscopy were performed in the CHI660e electrochemical workstation at a frequency of 100 kHz to 0.1 Hz and an oscillation potential of 10 mV. Composite electrolyte samples about ∼400 μm thick were sandwiched amid two metal sheet steels for the formation of test cells. The ionic conductivity can be expressed as follows:

1 *σ* = *L*/*R*_b_*S*

where *σ* refers to the ionic conductivity, *R*_b_ means the bulk resistance, *L* represents the thickness of electrolyte membranes, and *S* denotes the stainless-steel electrode area. All-solid-state lithium batteries adopted LiFePO_4_ as the cathode and lithium metal as the anode for assembly, and the corresponding charge–discharge and cycling performance were investigated by using the LANHE CT2001A device.

### 2.4 Theoretical calculation

The influence of the functional groups on ion conductivity of the system was calculated and analyzed by using the Vienna *ab initio* simulation package (VASP). The first-principles calculations under density functional theory (DFT) were carried out with the spin-polarized generalized gradient approximation (GGA). Core electron states were denoted by using the projector-augmented-wave technique applied by VASP.^29–31^ The exchange–correlation interactions were processed by using the GGA parameterized by Perdew, Burke, and Ernzerh (PBE) and represented by a plane wave with a wavefunction cutoff energy of 400 eV. The electronic wavefunction was converged to a tolerance of 10^−5^ eV (EDIFF = 10^−5^), whereas the geometric optimization tolerance was taken as 0.05 eV Å^−1^ (EDIFFG = −0.05). The calculation absorption energy of Li to the adsorbed [CH_2_OH]_*n*_ and [CH_2_OLi]_*n*_ is defined as follows:

2 Δ*E* = *E*([CH_2_OH]_*n*_/Li) − *E*(Li) − *E*([CH_2_OH]_*n*_)

3 Δ*E* = *E*([CH_2_OLi]_*n*_/Li) − *E*(Li) − *E*([CH_2_OLi]_*n*_)

## 3 Results and discussion

### 3.1 Synthesis routes of electrolytes PU/LiTFSI (SPE 1), PU/LiTFSI–Al_2_O_3_ (SPE 2), and PU/LiTFSI–Al_2_O_3_–LiOH (SPE 3)

**Scheme 1:**
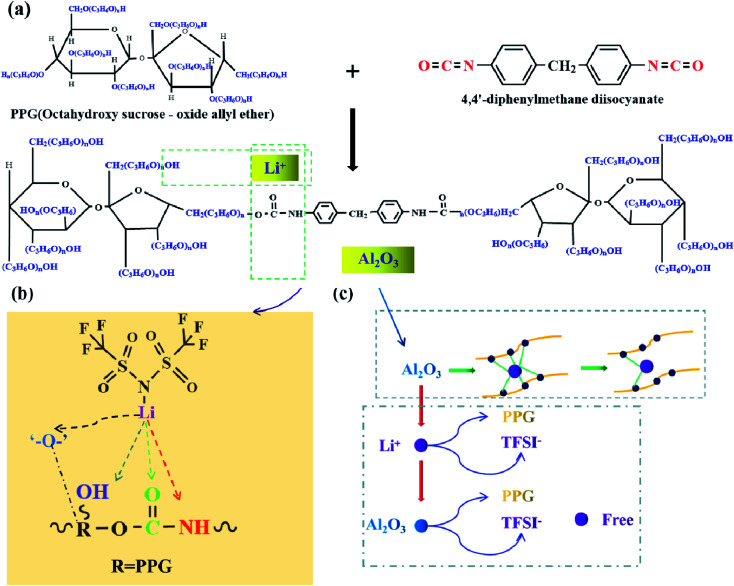
(a) The reaction formula of electrolytes SPE 1 and SPE 2; (b) positions of the action of Li^+^ in SPEs; (c) schematic diagram of the role of Al_2_O_3_ in the SPE of SPE 2.

Scheme 1 shows the reaction schematic of electrolytes PU/LiTFSI (SPE 1) and PU/LiTFSI–Al_2_O_3_ (1.3%) (SPE 2). The PU is mainly obtained by the reaction of “–OH” and “–NCO”. After LiTFSI is added into PPG, the electrolyte SPE 1 is formed by the reaction of PPG and MDI. And the electrolyte SPE 2 can be obtained by adding Al_2_O_3_ before it reacts with MDI. Meanwhile, according to Scheme 1(a) and (b), we can see that besides the bond of “–O–,” there are other three complex points of Li^+^ in the PU structure (“–C=O”, “–OH” and “–NH”).^21^Scheme 1(c) illustrates the role of Al_2_O_3_ in a polymer system. Al_2_O_3_ can not only reduce the coupling degree of the polymer with Li^+^ but also form an effective equilibrium system with the polymer and anionic groups, and thus increases the number of free Li^+^ in the system.^32^

**Scheme 2:**
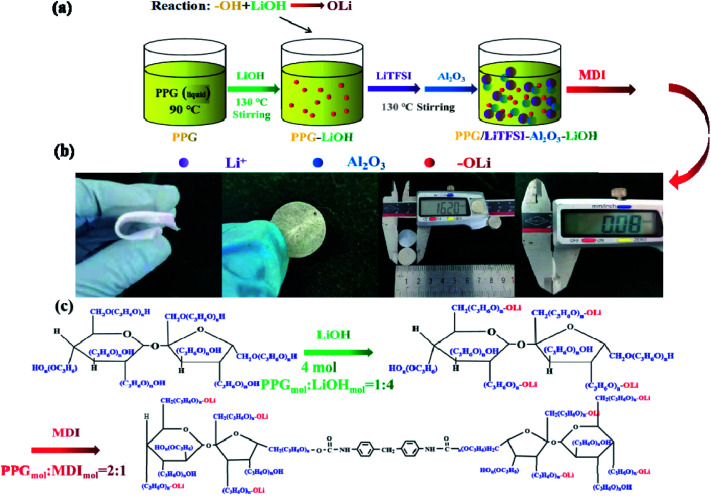
(a) Flow chart of preparation of the SPE 3; (b) physical diagram of SPE 3; (c) the reaction schematic of electrolyte the SPE 3.

Scheme 2 shows the preparation process and synthesis route of the electrolyte SPE 3. The PPG first reacts with LiOH (PPG_mol_ : LiOH_mol_ = 1 : 4) and stirring for 36 h at 130 °C in order to remove water (this step is to remove residual water from the system, prevent it from reacting with MDI and eliminate possible hydrolysis of the polyurethane), this step is to make functional group “–OH” into “–OLi”. Then LiTFSI and Al_2_O_3_ are added into system until completely dissolved. At last, the molar ratio of PPG to MDI was PPG_mol_ : MDI_mol_ = 2 : 1. It should be noted that after the final product is obtained, the product needs to be placed in a vacuum drying oven for 48 hours to remove the remaining water (water includes moisture in the air and produced during experiments).

### 3.2 Characterization of electrolyte SPE 3

**Fig. 1:**
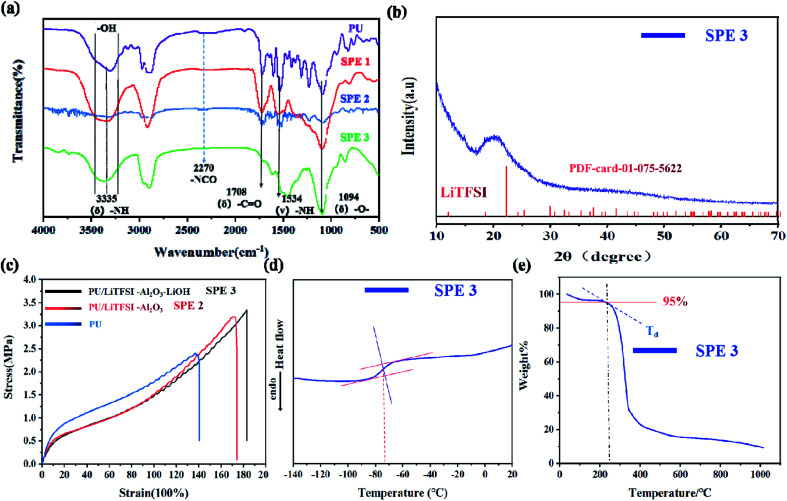
Characterization of electrolyte. (a) Infrared spectra of PU, SPE 1(PU/Li^+^), SPE 2 (PU/Li^+^-Al_2_O_3_) and SPE 3 (PU/Li^+^–Al_2_O_3_–LiOH); (b) XRD pattern; (c) the stress–strain curves of as-prepared PU, PU/LiTFSI–Al_2_O_3_(SPE 2), and PU/LiTFSI–Al_2_O_3_(1.3%)–LiOH(PPG_mol_ : LiOH_mol_ = 1 : 4) (SPE 3). (d) DSC curves of the SPE 3 membranes; (e) TGA curves of the SPE 3 membranes.

Fig. 1(a) shows infrared spectra of the electrolyte SPE 3. The peaks at 3335, 1708, and 1094 cm^−1^ correspond to the stretching vibration peaks at “–NH”, “–C=O” and “–C–O–C”, respectively.^33^ The peaks of 1534 and 1238 cm^−1^ are bending vibration peaks of “–NH” and vibration peaks of “–CN,” respectively. The characteristic peak of “–N=C=O” at 2270 cm^−1^ disappeared, indicating that the isocyanate has been reacted completely, which means that the PU has been prepared.^34^ Something to watch out for in the infrared spectrum, as can be seen from Fig. 1(a), the hydroxyl peak still exists, which proves that there are still unreacted “–OH” on the molecules.

After adding lithium salt in PU, the SPE 1 was obtained and the spectrum is more smooth due to the interaction between Li^+^ and the functional groups, which makes the vibration of polymer chain more regular. After the addition of Al_2_O_3_ in SPE 1, the peak intensity (SPE 2) of several functional groups decreases, indicating that inert filler can effectively reduce the coupling of the polymer chain of the electrolyte and the vibration will significantly decreases. After the addition of LiOH (SPE 3), the strong coupling effect of Al_2_O_3_ with the polymer chains has been moderated leading to the reappearance of the characteristic peaks. The ‘–OH’ peak was slightly shifted due to changes in some functional groups. As ‘–OH’ changes to ‘–OLi’, the hydroxyl peak intensifies. The main peak of OH splits into two peaks with the approximately equal intensity.

Fig. 1(b) shows the XRD pattern of the electrolyte SPE 3. There is a diffraction peak at 20°, which indicates the formation of ordered hydrogen bonds between and within molecules in polyurethane, so the existence of polyurethane structure can be further proved.^35,36^

The stress–strain curves of the PU, SPE 2, and SPE 3 films are illustrated in Fig. 1(c). The stress strength of pure PU was 2.4 MPa, and the relevant elongation-at-break value was 140%. After adding the LiTFSI and Al_2_O_3_, the stress strength increased significantly, reached 3.2 MPa, and the relevant elongation-at-break value reached 175%. After LiOH was added to PPG to complete the functional group modification, the stress strength slightly reached at 3.3 MPa, and the elongation-at-break value was 185%. The above characterization indicates that the PU and SPEs prepared show good stress strength properties, and their stress strain properties are higher than those of other SPEs reported.^37–39^

The thermal properties for the SPE 3 membranes were characterized by DSC and TGA. Fig. 1(d) shows the values of the glass transition temperature (*T*_g_) is about −73 °C which indicates the electrolyte has good flexibility at room temperature and its thermogravimetric analyses of the SPE 3 membranes are shown in Fig. 1(e). The degradation temperatures at a 5% weight loss (*T*_d_, 5%) of the SPE 3 is 234 °C. The electrolyte showed good thermal stability.

**Fig. 2:**
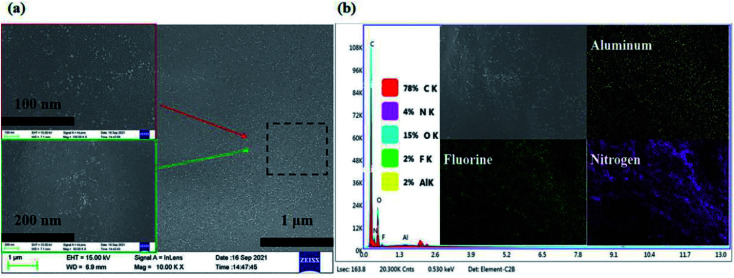
(a) The SEM image of SPE 3 and (b) the elements mapping of SPE 3.

Fig. 2(a) shows the SEM image and elements mapping of the electrolyte SPE 3. As can be seen from the Fig. 2(b), the Al_2_O_3_ nanoparticles and LiTFSI in polymer system are distributed on the system, which proves that the system exhibits good compatibility for the fillers added (Li element can't be mapped out, F element is LiTFSI).

### 3.3 Ionic conductivities of SPEs

**Fig. 3:**
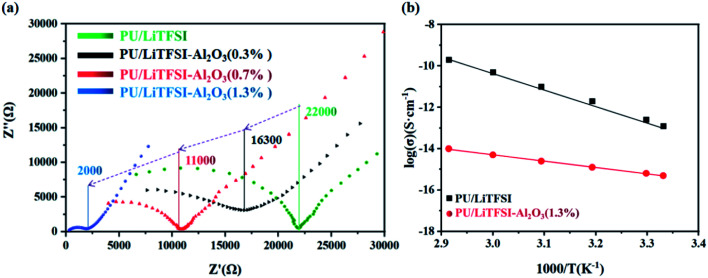
(a) AC impedance diagrams of the PU/LiTFSI–Al_2_O_3_ system with different Al_2_O_3_ dosages; (b) the Arrhenius plots for the ionic conductivities of SPE 1 and SPE 2.

Fig. 3(a) shows AC impedance diagrams of the PU/LiTFSI–Al_2_O_3_ electrolytes with Al_2_O_3_ amounts from 0 to 1.3%. It is found that Al_2_O_3_ dosage have a great impact on ion conductivity of the PU/LiTFSI–Al_2_O_3_ electrolytes. When the molar ratio of Al_2_O_3_ in the system is 1.3%, its conductivity increased from 2.1 × 10^−6^ S cm^−1^ to 2.5 × 10^−5^ S cm^−1^.

The ionic conductivities of the SPE 1 and SPE 2 at different temperatures were investigated and the Arrhenius plots for the ionic conductivities of the membranes are presented in Fig. 3(b). For the two kinds of SPEs, the Arrhenius plots of the ionic conductivity against the temperature is linear, indicating that the conductivity of the polymer electrolyte obeys Arrhenius law.^40^ It is also observed that the ionic conductivity increases with increasing temperature. The motion of polymer chains with the interaction or coordination of lithium ions decides the ionic conductivity.^41^ As the temperature increases, the quick movement of polyurethane chains leads to higher ionic conductivity.^21^

Table 1 listed the ion conductivity of the PU/LiTFSI–Al_2_O_3_ with different Al_2_O_3_ dosage. It can be seen that when the Al_2_O_3_ content exceeds the maximum 1.3%, the conductivity does not change.

**Table 1:** Ionic conductivity of PU/LiTFSI–Al_2_O_3_ system with different Al_2_O_3_ dosages

Samples	Mole ratio^a^ % (to system)	*L* b (thickness, μm)	*Z*′^c^ (Ω)	*S* = π*R*^2^^d^ (*R* = 0.5 cm)	Conductivity (S cm^−1^)
1	0.0	400	22 000	π/4	2.3 × 10^−6^
2	0.3	400	16 300	π/4	3.1 × 10^−6^
3	0.7	400	11 000	π/4	4.6 × 10^−6^
4	1.0	400	3750	π/4	1.4 × 10^−5^
5	1.3	400	2000	π/4	2.5 × 10^−5^
6	1.7	400	2000	π/4	2.5 × 10^−5^
7	2.0	400	2000	π/4	2.5 × 10^−5^

a For added system (0.03 mol) percentage.

b The sample thickness.

c Electrolyte impedance value.

d Electrolyte area.

**Fig. 4:**
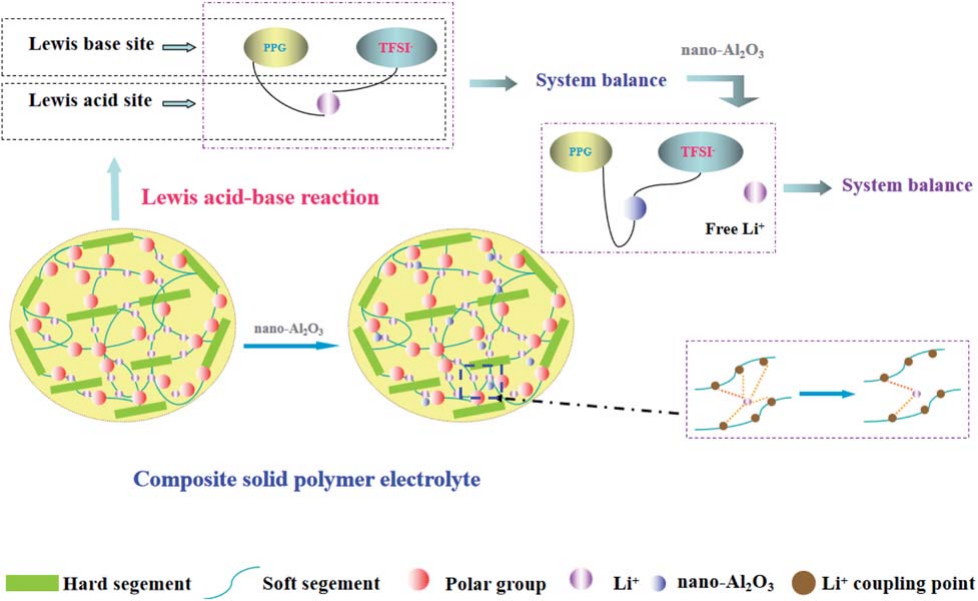
Schematic diagram of Al_2_O_3_ role in polymer electrolytes and acid-base theory.

It can be seen from the Table 1 that Al_2_O_3_ has a great influence on the conductivity of the system. It is possible that the high surface energy of the particles affects the conduction of nearby Li^+^.^42^ In addition, the Al_2_O_3_ content (1.3%) which we used was lower than other reports (10–20%), so a model based on the acid-base theory is proposed (Fig. 4) to explain it.

PPG is an amorphous polymer, in which the role of Al_2_O_3_ is only to reduce the coupling between Li^+^ and polymer chains, and there is no reduction in crystallization. Among acidic Al_2_O_3_, neutral Al_2_O_3_, and basic Al_2_O_3_, acidic Al_2_O_3_ shows the strongest effect on the polymer surface.^32^ Second, according to the acid-base theoretical model which proposed by Wieczorek^43^ and Croce,^32^ it is explained that the ionic conductivity of the system is related to the acid-base balance in the system. After adding LiTFSI into the polymer system, PPG and TFSI^−^ were used as the Lewis base while Li^+^ was used as the Lewis acid, and thus the system reached equilibrium. When Al_2_O_3_, as the Lewis acid, is added to the system, it takes precedence over Li^+^ to form an equilibrium system with PPG and TFSI^−^; meanwhile, a large number of free Li^+^ are released. When the addition amount of Al_2_O_3_ reaches a certain value, the system tends toward equilibrium and the number of free Li^+^ reaches the maximum value. As a result, the ionic conductivity of the system reaches the highest. However, when the amount of Al_2_O_3_ exceed the maximum value, the ionic conductivity of the system remains constant because the later added Al_2_O_3_ amount cannot form a new acid-base equilibrium with the system and thus cannot destroy the original equilibrium system.

Different from acidic-Al_2_O_3_, inert-Al_2_O_3_ (alkaline-Al_2_O_3_, α-Al_2_O_3_) mechanism of action is more reflected in the dosage. According to model proposed by WANG,^44^ only when the amount of alkaline-Al_2_O_3_ reaches a certain amount, the interaction between Al_2_O_3_ and hydrogen bond can form ion channels. So that explains why we use so much less than other systems.

**Fig. 5:**
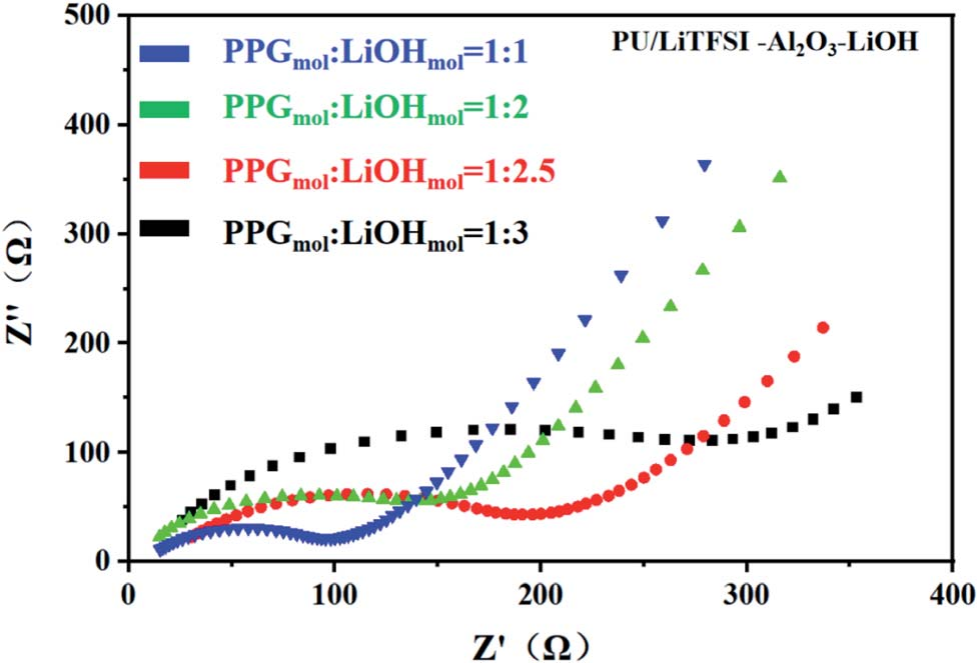
AC impedance for different LiOH dosages in PU/LiTFSI–Al_2_O_3_(1.3%)–LiOH system.

**Fig. 6:**
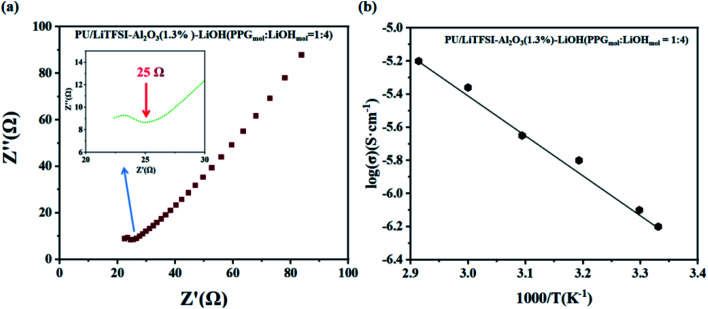
(a) AC impedance diagrams of SPE 3, (inset: the abscissa is not clear part); (b) the Arrhenius plots for the ionic conductivities of SPE 3.

Fig. 5 shows the AC impedance diagram of the PU/LiTFSI–Al_2_O_3_–LiOH system with different LiOH dosages. When the ratio of PPG to LiOH is PPG_mol_ : LiOH_mol_ = 1 : 3, the ion conductivity reaches 1.7 × 10^−4^ S cm^−1^. As we can see from Fig. 6(a), with the increase of the addition amount of LiOH, the ion conductivity reaches the 2 × 10^−3^ S cm^−1^ at the ratio of PPG_mol_ : LiOH_mol_ = 1 : 4. And from the Fig. 6(b), we know that the Arrhenius plots of the ionic conductivity against the temperature is linear, indicating that the conductivity of the polymer electrolyte obeys Arrhenius law.

It can be seen from the Table 2 that the addition amount of LiOH has a great influence on the system. The improvement of ionic conductivity is mainly attributed to the reaction between LiOH and PPG, which leads to the change of partial “–OH” into “–OLi”.

**Table 2:** Ionic conductivity of PU/LiTFSI–Al_2_O_3_–*x*LiOH (*x* = 1, 2, 2.5, 3, 4, 4.5, 5) system

Samples	PPG_mol_ : LiOH_mol_	*L* aa (thickness, μm)	*Z*′^b^ (Ω)	*S* = π*R*^2^^c^ (*R* = 0.5 cm)	Conductivity (S cm^−1^)
1	1 : 1.0	400	300	π/4	1.7 × 10^−4^
2	1 : 2.0	400	200	π/4	2.5 × 10^−4^
3	1 : 2.5	400	150	π/4	3.4 × 10^−4^
4	1 : 3.0	400	100	π/4	5.0 × 10^−4^
5	1 : 4.0	400	25	π/4	2.0 × 10^−3^
6	1 : 4.5	400	25	π/4	2.0 × 10^−3^
7	1 : 5	400	25	π/4	2.0 × 10^−3^

a The samples thickness.

b Electrolyte impedance value.

c Electrolyte area.

**Fig. 7:**
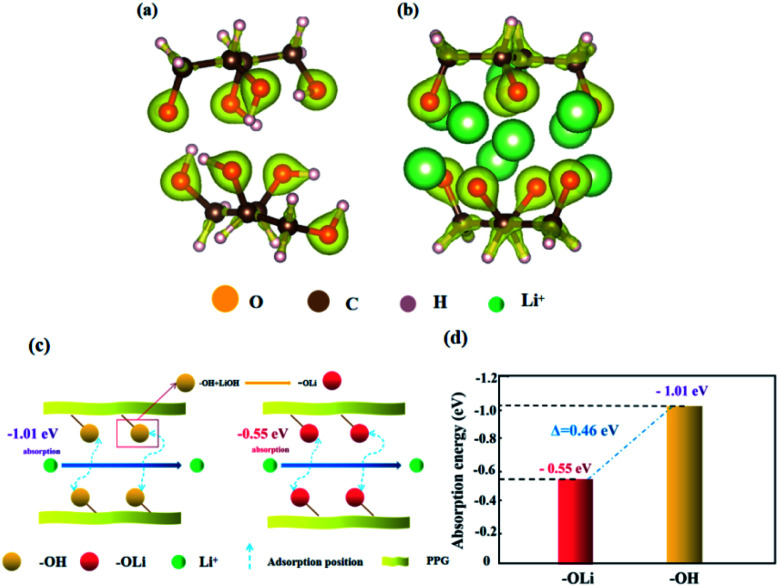
(a and b) Adsorption model of Li^+^ on “OH” and “OLi” functional groups by VASP; (c) the effects of “OH” and “OLi” on the migration of lithium ions; (d) diagram of the adsorption energy of Li^+^ on “OH” and “OLi”.

Fig. 7 is the adsorption models of functional groups “OH” and “OLi” on Li^+^. Based on the change of functional groups, we demonstrate the effect of the change of functional groups on the conductivity from the perspective of mechanism and theoretical calculation.

First, after the change of “OH” into “OLi”, the electron cloud is mainly distributed around O atoms, which reduces the free electrons in the middle of the space charge layer and provides more favorable conditions for ion transport (Fig. 7(a) and (b)). Under the action of the applied electric field and intramolecular electrostatic interaction^45^ force, it speeds up ion transport. Thus, the ionic conductivity increases.

Second, the absorption energies of “OLi/Li^+^” and “OH/Li^+^” obtained by density functional theory (DFT) calculation were −0.55 eV and −1.01 eV respectively, which indicates that both functional groups can generate spontaneous adsorption of Li^+^. According to Fig. 6(c) and (d), it can be concluded that the adsorption energy of “OLi/Li^+^” is far less than that of “OH/Li^+^” on Li^+^(Δ*E*_“OH/Li+”–“OLi/Li+”_ = 0.46 eV), which indicates that Li^+^ is easier to desorb from the “OLi” group, thus improving the overall ion conductivity of the system.

### 3.4 Performance of LFP|SPE|Li batteries

**Fig. 8:**
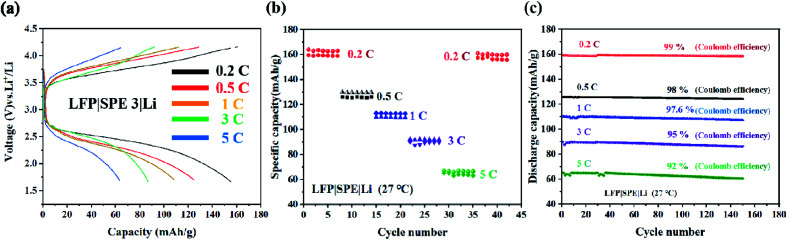
LFP|SPE 3|Li batteries. (a) The first charge/discharge curves at ambient temperatures for 0.2C, 0.5C, 1C, and 5C; (b) rate capacity with various rates; (c) the cycling properties provided with different rates.

We assemble the LFP (10 μm) |SPE 3 (80 μm) |Li (250 μm) battery in order to study the application of the electrolyte in the battery device performance. Fig. 8(a) exhibits the specific capacity of LFP|SPE 3|Li batteries with different cycle rates. The capacity reached 159.6 mA h g^−1^ at 0.2C, quite approximating the theoretical specific capacity (170 mA h g^−1^) in LFP cathode materials. This proves the favorable dynamic process and Li^+^ conducting path of solid-state LFP|SPE 3|Li batteries. According to Fig. 8(b) and (c), it can be found that although the retention of the battery capacity decayed from 0.2C to 5C, the Coulomb efficiency remained stable at 90–98% at different rates and capacity retention are 99%, 98%, 97%, 95% and 92% respectively at different rates. All the results show that the performance of the cell prepared by us is superior to the previous reports.^21,26^ The cyclic stability of the LFP|SPE|Li battery under different rates is attributed to the highly ionic conductivity of the SPE 3 film, revealing the huge potentials of the SPE 3 in improving lithium battery industrialization.

**Fig. 9:**
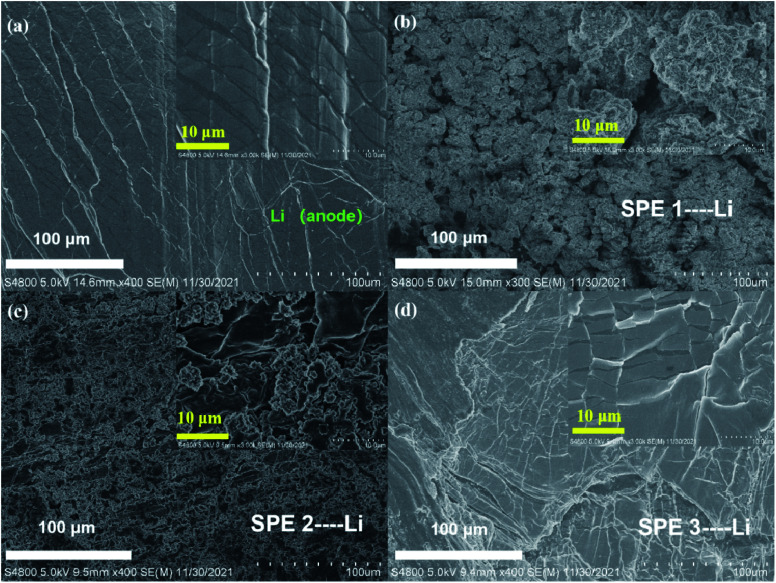
SEM images of lithium anode surface and were obtained after 100 cycles under 0.5C conditions. (a) Uncirculated lithium anode, new lithium sheet; (b) lithium anode surface under SPE 1 condition; (c)lithium anode surface under SPE 2 condition; (d)lithium anode surface under SPE 3 condition.

Fig. 9(a)–(d). show the SEM images of pristine Li metal anode and three Li metal anode after 100 cycles against with different SPEs. Fig. 9b indicates the surface of the Li metal anode is quite tough which indicates the uneven Li deposition and poor stability. This phenomenon mainly comes from the poor inherent ion conductivity of SPE 1 (2.3 × 10^−6^ S cm^−1^). With the enhancement of the conductivity of the SPE 2 sample (2.5 × 10^−5^ S cm^−1^), the deposition of Li becomes a bit more even (Fig. 9c). By contrast, as the ion conductivity reaches 2 × 10^−3^ S cm^−1^ (SPE 3), the cycled Li anode remains smooth Fig. 9d due to the ultra-even deposition. All these results demonstrate that the inherent high-conductivity of SPE can significantly release the uneven Li deposition which contributes to the stability of Li metal anode.

**Fig. 10:**
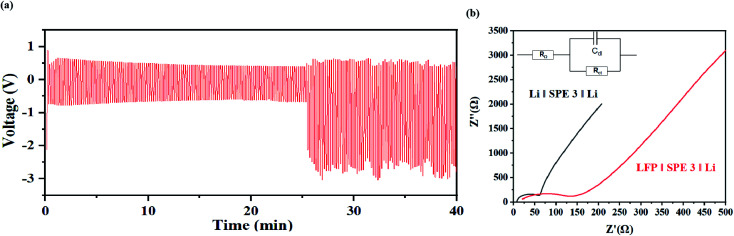
(a) Voltage–time curve of the Li|SPE 3|Li at the current density of 1 mA cm^−1−2^. (b) AC impedance before and after the battery cycle.

From Fig. 10(a), during the charge discharge cycle, the voltage change of each cycle is similar, and in the initial stage of the cycle, the continuous decrease of the average voltage may come from the decrease of overpotential caused by electrode activation. As shown in Fig. 10(a), in the initial stage of the cycle of the symmetrical battery, there is a gradually decreasing overpotential, but the overall shape of the voltage signal remains consistent. When the charging or discharging is almost over, there is an obvious voltage rise signal due to polarization. The polarization voltage after 25 min may mainly come from the high interface impedance between the SPE and the lithium metal (Fig. 10(b)). Due the surface passivation of lithium metal at high potential, a tough metal surface is generated which results in the decrease of the active surface area. This process may significantly sluggish the ion-transport kinetics and an extra potential is need to drive the deintercalation of Li+ and cover the high diffusion energy-barrier on the interface. This problem can be solved by adding a buffer layer to stable the dissolution and deposition of Li^+^ on the surface, which will be study in our future work.

## 4 Conclusions

In summary, a new composite polymer electrolyte system based on polyurethane/lithium salt/Al_2_O_3_/LiOH was prepared. By optimizing addition rate of LiTFSI, Al_2_O_3_, and LiOH, the ion conductivity of PU/LiTFSI–Al_2_O_3_(1.3%)–LiOH (PPG_mol_ : LiOH_mol_ = 1 : 4) was increased to 2 × 10^−3^ S cm^−1^ at room temperature. The battery displays excellent cycling and electrochemical properties. The specific discharge capacity was around 159.6 mA h g^−1^ at 0.2C, much approximating the theoretical specific capacity (170 mA h g^−1^) in LFP cathode materials, and the Coulomb efficiency was found to be stable in the region of 92–98% at five different rates. This work will provide a theoretical basis and experimental data for the preparation of composite polymer electrolytes in the near future.

## Conflicts of interest

There are no conflicts to declare.

## Supplementary Material
